# Psychometric properties of the Chinese version of the Pittsburgh Sleep Quality Index (PSQI) among Hong Kong Chinese childhood cancer survivors

**DOI:** 10.1186/s12955-021-01803-y

**Published:** 2021-07-06

**Authors:** K. Y. Ho, Katherine K. W. Lam, W. Xia, J. O. K. Chung, Ankie T. Cheung, Laurie L. K. Ho, S. Y. Chiu, Godfrey C. F. Chan, William H. C. Li

**Affiliations:** 1 School of Nursing, Hong Kong Polytechnic University, Hung Hom, Hong Kong, SAR; 2 School of Nursing, Sun Yan-Sen University of Medical Sciences, Guangzhou, China; 3 School of Nursing, University of Hong Kong, Pok Fu Lam, Hong Kong, SAR; 4 Hong Kong Children’s Hospital, Kowloon Bay, Hong Kong, SAR

**Keywords:** Sleep disruption, Childhood cancer survivors, Assessment, Psychometric properties

## Abstract

**Background:**

Sleep disruption is a prevalent symptom reported by survivors of childhood cancer. However, there is no validated instrument for assessing this symptom in this population group. To bridge the literature gap, this study translated and adapted the Pittsburgh Sleep Quality Index (PSQI) for Hong Kong Chinese cancer survivors and examined its psychometric properties and factor structure.

**Methods:**

A convenience sample of 402 Hong Kong Chinese childhood cancer survivors aged 6–18 years were asked to complete the Chinese version of the PSQI, Center for Epidemiologic Studies Depression Scale for Children (CES-DC), Fatigue Scale-Child (FS-C)/Fatigue Scale-Adolescent (FS-A), and Pediatric Quality of Life Inventory (PedsQL). To assess known-group validity, 50 pediatric cancer patients and 50 healthy counterparts were recruited. A sample of 40 children were invited to respond by phone to the PSQI 2 weeks later to assess test–retest reliability. A cutoff score for the translated PSQI used with the survivors was determined using receiver operating characteristic analysis.

**Results:**

The Chinese version of the PSQI had a Cronbach alpha of 0.71, with an intraclass correlation coefficient of 0.90. Childhood cancer survivors showed significantly lower mean PSQI scores than children with cancer, and significantly higher mean scores than healthy counterparts. This reflected that childhood cancer survivors had a better sleep quality than children with cancer, but a poorer sleep quality than healthy counterparts. We observed positive correlations between PSQI and CES-DC scores and between PSQI and FS-A/FS-C scores, but a negative correlation between PSQI and PedsQL scores. The results supported that the Chinese version of the PSQI showed convergent validity. Confirmatory factor analysis showed that the translated PSQI data best fit a three-factor model. The best cutoff score to detect insomnia was 5, with a sensitivity of 0.81 and specificity of 0.70.

**Conclusion:**

The Chinese version of the PSQI is a reliable and valid instrument to assess subjective sleep quality among Hong Kong Chinese childhood cancer survivors. The validated PSQI could be used in clinical settings to provide early assessments for sleep disruption. Appropriate interventions can therefore be provided to minimize its associated long-term healthcare cost.

*Trial registration* This study was registered in ClinicalTrials.gov with the reference number NCT03858218.

## Background

Cancer is a major cause of death in the pediatric population. In Hong Kong, 46 patients aged 0–19 years died from cancer in 2016 [1]. According to the Hong Kong Cancer Registry, approximately 190 new cases of childhood cancer are diagnosed each year [2]. Although this figure is lower than for adults, childhood cancer remains a significant healthcare concern because of its damaging effects on children’s physical and psychological functions throughout life [3]. Particularly, children surviving cancer still have to bear the health burden of numerous sequelae, even if their treatment finished months or even years previously [4].

One of the most prevalent symptoms reported by patients who survive cancer is sleep disruption [5]. It is defined as a range of difficulties in achieving enough good quality sleep [6]. One systematic review reported that 25% to 59% of adults report sleep disruption after cancer treatment [5]. A retrospective cohort study found that 16.7% of adult survivors of childhood cancer complained of disrupted sleep [7]. The causes of sleep disruption are multifactorial [5]. However, it can largely attributed to disruption of circadian rhythm by prolonged use of medications and long-term hospitalization [5]. Sleep disruption can have severe negative effects on survivors of cancer [8]; it precipitates inflammation and oxidative stress, which contribute to neurocognitive impairment [9]. Sleep disruption can also lead to fatigue [10], which limits patients’ capacity to engage in daily activities [11] and results in depression [12], severely compromising patients’ long-term quality of life [13]. Therefore, healthcare professionals should recognize their responsibility to implement appropriate interventions to improve cancer survivors’ sleep quality and minimize the associated health consequences of sleep disruption. The development and evaluation of such interventions requires a reliable and valid instrument that can precisely assess the sleep quality of cancer survivors.

The Pittsburgh Sleep Quality Index (PSQI) is the most commonly used instrument to assess the subjective sleep quality of adults in clinical and community settings [14]. Although this instrument was originally developed for psychiatric patients [15], it has been validated in different populations of adult cancer patients [16, 17] and extensively applied in studies of this population [14]. Increasingly, the PSQI has been used to assess children’s sleep quality [18]; there is evidence that it is a reliable and valid instrument for young people [19], community-based adolescents [20], and college students [21]. However, the PSQI has never been validated for use with pediatric patients, including childhood cancer survivors [22]. Since cancer and its treatment pose a significant impact on sleep, the symptom presentation of sleep disruption in childhood cancer survivors may be different when compared to that in healthy children [23]. Given the above issue, it may not be appropriate to apply the PSQI to assess the sleep quality of childhood cancer survivors before confirming its psychometric properties in this population. A review of the literature revealed that no validated instrument is available for assessing sleep quality in childhood cancer survivors. This lack of validated instruments precludes understanding the severity of the problem and hinders the development of appropriate interventions to promote sleep quality. This study aimed to translate and adapt the Pittsburgh Sleep Quality Index (PSQI) for Hong Kong Chinese cancer survivors and examine its psychometric properties and factor structure.

The convergent validity of PSQI was established by identifying correlations between sleep quality and related variables (i.e. depressive symptoms, fatigue and quality of life) as measured by validated scales. Following previous studies [10, 12, 13], we hypothesized positive correlations between sleep quality and depressive symptoms and between sleep quality and fatigue, but a negative correlation between sleep quality and quality of life. In addition, previous literature indicated that most adverse effects of cancer treatment will gradually subside once treatment is discontinued [24, 25]. Therefore, to establish the discriminant validity, it is hypothesized that childhood cancer survivors would report better sleep quality than children with cancer. Whereas, due to long-lasting impact of cancer and its treatment, childhood cancer survivors would report poorer sleep quality than healthy counterparts [23]. Concerning the factor structure of the PSQI, [15, 26, 27], results vary according to sample characteristics [15, 26, 27]. Although Buysse et al.[15] suggested a single-factor model underlying the PSQI in depressive patients, Cole et al. [26] proposed a three-factor model in older adults and Magee et al. [27] identified a two-factor model in Australian adults. As such, confirmatory factor analysis was conducted to compare the overall fits of the single-factor, two-factor, and three-factor models underlying the Chinese version of the PSQI.

## Methods

### Design

A cross-sectional study was conducted to validate the Chinese version of the PSQI. The study was carried out in the pediatric oncology outpatient clinic of Queen Mary Hospital in Hong Kong. This clinic provided medical consultation for a majority of childhood cancer survivors in Hong Kong. Hence, our sample was representative to the total population [28].

### Sample/participants

Hong Kong Chinese childhood cancer survivors who had outpatient medical follow-ups were invited. Eligible survivors were aged 6–18 years, and able to communicate in Cantonese and read Chinese. We excluded survivors whose medical records identified cognitive or behavioral problems. To ensure that all participants could understand the questionnaire, we included only those older than 6 years.

To assess the known-group validity, 50 pediatric cancer patients and 50 healthy counterparts were recruited using the same inclusion and exclusion criteria.

Childhood cancer survivors were defined as children who have completed all cancer treatment [29], while children with cancer were those who received active treatment for cancer [30]. Healthy children were those who reported without any chronic disease [31].

There is no universal guideline on the minimal sample size required for confirmatory factor analysis. Nunnally [32] suggested that at least 10 subjects are required for each item. As the PSQI contains 19 items, we planned to recruit at least 380 childhood cancer survivors.

### Translation

An expert panel was set up for translation. The panel contained an associate professor, two assistant professors, a research assistant professor, a postdoctoral fellow, a pediatric oncologist, and a nurse specialist. The procedures suggested by Bracken and Barona [33] were followed. The PSQI was translated from English into Chinese by two independent bilingual translators. The two translations were compared and reconciled after panel member discussions. Another two independent bilingual translators blinded to the original English version of the scale were asked to translate the Chinese version back into English. The panel members compared the back translations against the original English version and decided whether the meaning of each item in the back translations had been retained. Disagreement was resolved during regular meetings.

### Instrument

#### Demographic and clinical characteristics

A structured questionnaire was used to record participant demographic and clinical characteristics.

#### The Pittsburgh Sleep Quality Index (PSQI)

The 19-item PSQI assesses subjective sleep quality in the last month. Items are categorized into seven components: subjective sleep quality, sleep latency, sleep duration, habitual sleep efficiency, sleep disturbances, sleep medication, and daytime dysfunction. The possible score range for each component is 0 (no difficulty) to 3 (severe difficulty). The seven component scores are summed to produce a global score; higher scores represent poorer subjective sleep quality. Previous psychometric studies of the English version of the PSQI have confirmed that it is a reliable and valid instrument for assessing subjective sleep quality among adult cancer survivors [34].

#### The Chinese version of the Center for Epidemiologic Studies Depression Scale for Children (CES-DC)

The CES-DC assesses depressive symptoms in people aged 6–17 years. It comprises 20 items measured on a 4-point scale. Higher scores represent more depressive symptoms. This is a reliable and valid instrument for Hong Kong Chinese children [35].

#### The Chinese version of the Fatigue Scale-Child (FS-C)/Fatigue Scale-Adolescent (FS-A)

These two scales measure cancer-related fatigue levels in pediatric oncology patients. The FS-C comprises 13 items for patients aged 7–12 years; the FS-A contains 12 items for patients aged 13–18 years. Higher scores represent higher levels of cancer-related fatigue. They are reliable and valid measures of cancer-related fatigue among Hong Kong Chinese childhood cancer survivors [36, 37].

#### The Chinese version of the Pediatric Quality of Life Inventory 4.0 Generic Core Scale (PedsQL 4.0)

The PedsQL assesses health-related quality of life of people aged 2–18 years. This scale comprises 23 items categorized into four different subscales: physical functioning, emotional functioning, social functioning, and school functioning. Subscale scores are obtained by averaging item scores for the corresponding subscales; the total scale score is the average of all item scores. Higher PedsQL scores indicate better quality of life. The psychometric properties of this scale among Chinese children have been well established [38].

### Data collection

A research assistant approached all parents who accompanied their children for medical consultation in the outpatient clinic, and introduced the study. After checking eligibility, the research assistant obtained written consent from parents who allowed their child to participate. These parents were ensured that withdrawal would not result in any prejudice to the care received. The children were also invited to write their names on a child assent form to indicate their willingness to participate. The same procedures were used to identify and recruit eligible children with cancer in a pediatric oncology ward. As for healthy children, the recruitment was conducted in a community center in Hong Kong. A poster with study’s details was put on a notice board of the center. Parents who allowed their children to join could approach the center in-charge to indicate their willingness. Written parental consent was obtained by the in-charge after screening the eligibility. Healthy children were also invited to complete the child assent form.

After the informed consent process, data collection was performed in the outpatient clinic and community center. Parents were invited to complete a simple questionnaire that documented their child’s demographic and clinical characteristics (if applicable). Children were asked to self-complete the PSQI, CES-DC, FS-A/FS-C, and PedsQL without parental guidance. However, the research assistant who performed data collection were trained by our research team to provide further explanation for children in case they were in doubt. The whole process of data collection was around 15 min. No adverse feedback was received from children and parents. To assess test–retest reliability, a sample of 40 children were invited to respond by phone to the PSQI 2 weeks later.

### Data analysis

For semantic equivalence, the expert panel were asked to compare the original and translated versions of the PSQI, and rate the equivalence of each translated item using a 4-point scale (from 1 = not equivalent to 4 = most equivalent). The equivalence rate was calculated as the percentage of items rated as either 3 or 4. Any item rated 1 or 2 by 20% of the experts was deemed not equivalent and was amended.

For content equivalence, the expert panel rated the relevance of each PSQI item to the concept (i.e., subjective sleep quality) using a 4-point scale (from 1 = not relevant to 4 = very relevant). The content validity index (CVI) of an item (I-CVI) was calculated as the percentage of experts who rated the item as either 3 or 4. A CVI of the translated PSQI (S-CVI) was the average of the I-CVIs for all items. An I-CVI ≥ 0.78 or higher and S-CVI ≥ 0.9 were considered acceptable [39].

The internal consistency of the Chinese version of the PSQI was evaluated using Cronbach’s alpha, and the test–retest reliability using the intraclass correlation coefficient (ICC).

Convergent validity was established by identifying correlations between PSQI and CES-DC scores, between PSQI and FS-A/FS-C scores, and between PSQI and PedsQL scores.

Confirmatory factor analysis was performed. The overall fits of the single-factor, two-factor, and three-factor models were examined and compared because previous studies report varying PSQI factor structures [15, 26, 27]. The overall model fit was determined using following fit indices: the χ^2^/degrees of freedom (*df*) ratio, root mean square error of approximation (RMSEA), and comparative fix index (CFI). The χ^2^/ df ratio measures global fit and values between 1 and 5 indicate a good fit [40]. The RMSEA indicates model fit based on the population discrepancy function, with the value < 0.05 showing superior model fit [41, 42]. Despite a value of less than 0.05 in RMSEA is generally recommended to represent superior fit for a model [43], there is evidence that a value up to 0.08 in RMSEA suggests reasonable fit [42]. The comparative fit index indicates the degree of model fit compared with an independence model. It ranges from 0 to 1, with values ≥ 0.95 representing good fit [44].

A cutoff score for the translated PSQI used with the survivors was determined using receiver operating characteristic (ROC) analysis. Participants were regarded as having insomnia if they met all diagnostic criteria for insomnia in Diagnostic and Statistic Manual of Mental Disorders Fifth Edition (DSM-5). The area under the curve (AUC) was calculated. Sensitivity and specificity were used to determine the best cutoff score.

## Results

### Demographics

Table 1 shows participant demographics. The mean age of the survivors was 12.3 years (standard deviation = 3.8). Of patients, 55.7% (n = 224) were boys and 77.6% (n = 312) of children’s parents had upper secondary education or above. The types of cancer included leukemia, lymphoma, brain tumor, osteosarcoma, kidney tumor and germ-cell tumor. However, some categories had very small counts which might not fulfill statistical assumptions for data analysis. We therefore re-grouped the variable into 2 categories that is solid and non-solid tumors. Of patients, 66.9% (n = 269) were diagnosed with non-solid tumor, 58.2% (n = 234) had received only one type of treatment, and 57.3% (n = 230) completed their treatment less than 5 years ago. Comparative statistics indicated that the three groups were similar in age, sex distribution, and parental educational attainment.

**Table 1:** Demographic characteristics of the subjects (*N* = 502)

	n (%)				
	Childhood cancer survivors n = 402	Children with cancern = 50	Healthy children n = 50	x^2^/F	*p* value
*Sex*				1.82	0.40
Male	224 (55.7)	26 (52.0)	23 (46.0)		
Female	178 (44.3)	24 (48.0)	27 (54.0)		
*Parents’ Educational Attainment*				4.24	0.12
Lower secondary school or below	90 (22.4)	15 (30.0)	17 (34.0)		
Upper secondary school or above	312 (77.6)	35 (70.0)	33 (66.0)		
*Diagnosis*				1.70*	0.75*
Non-solid tumor	269 (66.9)	32 (64.0)	–		
Solid tumor	133 (33.1)	18 (36.0)	–		
Treatment received				0.70*	0.45*
One type of treatment	234 (58.2)	26 (52.0)	–		
More than one type of treatment	168 (41.8)	24 (748.0)	–		
*Age*				1.10	0.58
6–12 years	204 (50.7)	27 (54.0)	22 (44.0)		
13–18 years	198 (49.3)	23 (46.0)	28 (56.0)		
	*Mean (SD)*				
	12.3 (3.78)	11.8 (3.47)	12.2 (3.59)		0.63
*Time since treatment completed*					
Less than 5 year	230 (57.3)	–	–		
5 to 10 years	124 (30.8)	–	–		
More than 10 years	48 (11.9)	–	–		

^*^Significant at p < 0.05

### Validity

#### Semantic equivalence

The semantic equivalence ranged from 85.7%–100%. The overall rate was 99.2%, indicating that all items of the Chinese version of the PSQI were conceptually and idiomatically equivalent to those in the English version.

#### Content equivalence

Regarding the I-CVIs, the ratings ranged from 14.3%–100%, with an S-CVI of 95.2%, indicating that most items, except item 8, reflected the underlying construct. Item 8 was then revised. The recalculated S-CVI and I-CVI were 100%, confirming the content validity.

#### Construct validity

Table 2 shows the results of one-way between-subjects analysis of variance and post-hoc testing on PSQI scores for the three groups. Survivors of childhood cancer reported a significantly lower mean PSQI score than children with cancer (4.64 vs 6.60, *p* < 0.05), but a significantly higher mean score than their healthy counterparts (4.64 vs 3.38, *p* < 0.05). This confirmed the known-group validity.

**Table 2:** The test results of ANOVA on the levels of fatigue among the three groups

	Mean (SD)			G1 vs G2		G1 vs G3		G2 vs G3	
	G1	G2	G3	Mean difference	*p* value	Mean difference	*p* value	Mean difference	*p* value
Sleep quality	4.64	6.60	3.38	−1.96	0.00*	−1.26	0.00*	3.22	0.00*

*Significant at *p* < 0.05

SD = standard deviation; G1 = children who had survived cancer; G2 = children receiving treatment; G3 = healthy counterparts; with each group contained 50 subjects

Interrelationships between PSQI, CES-DC, FS-C/FS-A, and PedsQL scores among survivors were examined using the Pearson correlation coefficient. Correlation coefficients of 0.10–0.29, 0.30–0.49, and 0.50–1.0 can be interpreted as small, medium, and large, respectively [45]. For survivors aged 6–12 years (Table 3), there was a large positive correlation between PSQI and FS-C scores (r = 0.60, n = 204, *p* < 0.01), and between PSQI and CES-DC scores (r = 0.57, n = 204, *p* < 0.01), and a medium negative correlation between PSQI and PedsQL scores (r =  − 0.43, n = 204, *p* < 0.01). Survivors aged 13–18 years (Table 3) showed a strong positive correlation between PSQI and CES-DC scores (r = 0.64, n = 198, *p* < 0.01), a small positive correlation between PSQI and FS-A scores (r = 0.27, n = 198, *p* < 0.01), and a medium negative correlation between PSQI and PedsQL scores (r =  − 0.45, n = 198, *p* < 0.01). This indicated that survivors of childhood cancer who reported more sleep disturbance had more depressive symptoms and cancer-related fatigue and lower quality of life. Construct validity was demonstrated.

**Table 3:** Interrelationships between the scores of PSQI, CES-DC, FS-C and PedsQL among the survivors aged 6–12 years (n = 204) and survivors aged 13–18 years (n = 198)

Survivors aged 6–12 years (n = 204)				
	Global PSQI	CES-DC	FS-C	PedsQL
Global PSQI	–	0.57**	0.60**	−0.43**
CES-DC	0.57**	–	0.55**	−0.32**
FS-C	0.60**	0.55**	–	−0.31**
PedsQL	−0.43**	−0.32**	−0.31**	–

Survivors aged 13–18 years (n = 198)				
	Global PSQI	CES−DC	FS−A	PedsQL
Global PSQI	–	0.64**	0.27**	−0.45**
CES-DC	0.64**	–	0.32**	−0.57**
FS-A	0.27**	0.32**	–	−0.37**
PedsQL	−0.45**	−0.57**	−0.37**	–

CES-DC, Center for Epidemiologic Studies Depression Scale for Children; FS-A, Fatigue Scale-Adolescent; FS-C, Fatigue Scale-Child; PedsQL, Pediatric Quality of Life Inventory; PSQI, Pittsburgh Sleep Quality Index

**p value < 0.01

#### Confirmatory factor analysis

**Fig. 1:**
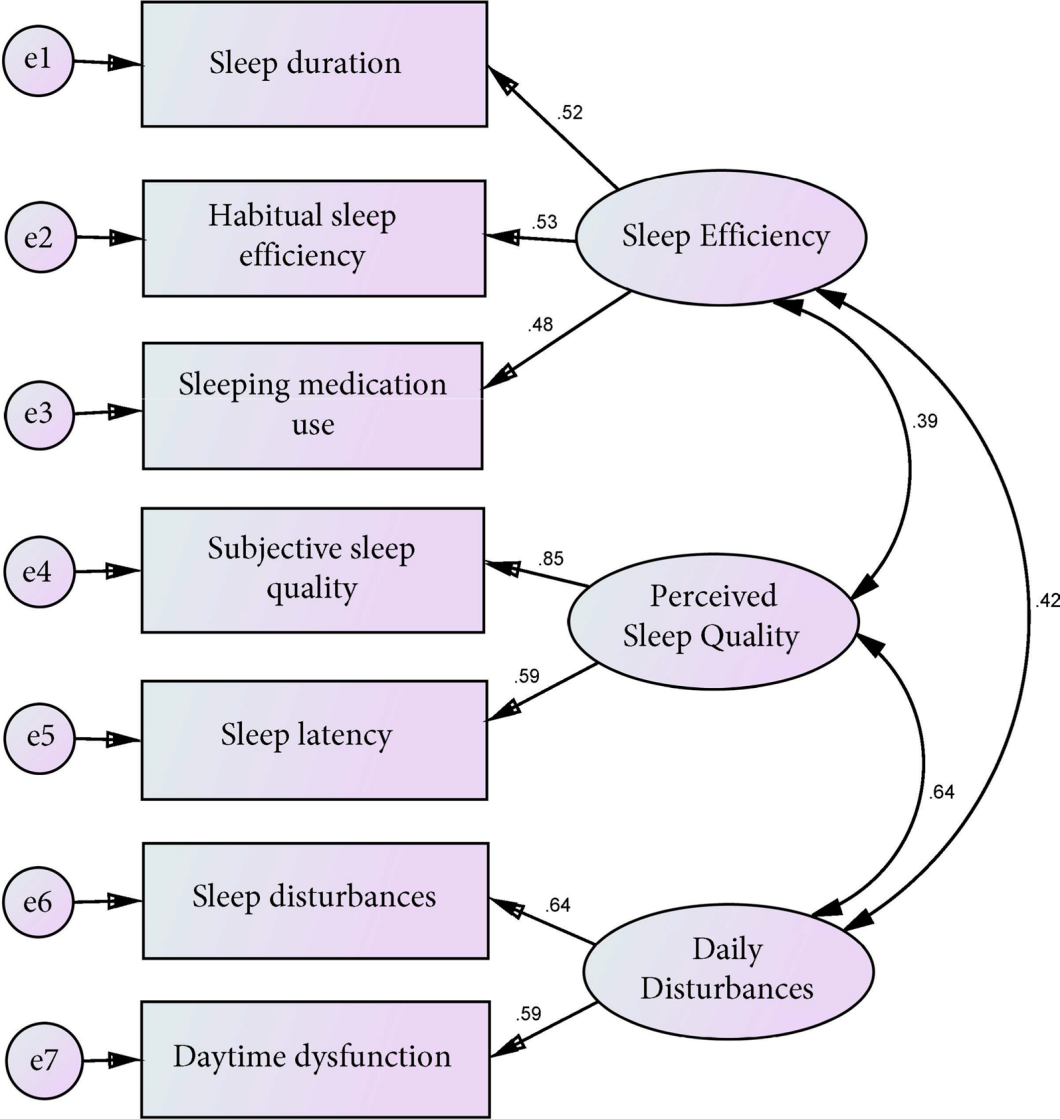
The estimated parameters of the Chinese version of the PSQI based on the three-factor model

Table 4 presents the fit indices of the Chinese version of the PSQI based on the single-factor, two-factor, and three-factor models. The results indicated that the seven component scores best fit the three-factor model. Figure 1 shows the estimated parameters of the Chinese version of the PSQI based on the three-factor model. All correlation matrices were positive and less than 1, thus were reasonable. Additionally, the factor loadings were high, ranging from 0.48 to 0.85. The t-values were greater than 2 and statistically significant. The standard errors were between 0.15 and 0.46, suggesting that all parameters were accurately estimated.

**Table 4:** Fit statistics for the Chinese version of the PSQI

Factor model	*x*^*2*^/df	CFI	RMSEA
1-factor model	10.36	0.62	0.15
2-factor model	7.21	0.78	0.12
3-factor model	3.12	0.94	0.08

*x*^*2*^/df, Relative chi-square; CFI, Comparative fix index; RMSEA, Root Mean Square Error of Approximation

Confirmatory factor analysis (CFA) was carried out using AMOS version 25.0 for Windows. Acceptable overall fit of each model was evaluated using the following indices:

**Table:** 

Criterion	Range
*x*^*2*^/df	1.00–5.00
CFI	0.9 or higher
RMSEA	0.08 or less

#### Reliability

The ICC at the 2-week interval was 0.90 (*p* < 0.001), which is higher than the cutoff of 0.7, indicating that the Chinese version of the PSQI is acceptable for research. The seven component scores of the translated PSQI achieved a Cronbach alpha of 0.71. The corrected item–total correlations ranged from 0.27 to 0.63, indicating acceptable internal consistency.

#### ROC analysis

**Fig. 2:**
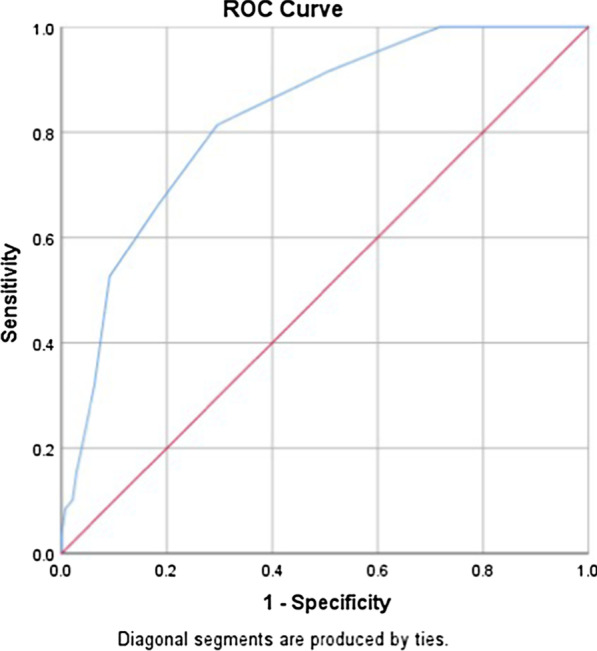
The AUC of the Chinese version of the PSQI when compared with the DSM-5 diagnostic criteria for insomnia

When compared with the DSM-5 diagnostic criteria for insomnia, the Chinese version of the PSQI demonstrated acceptable discrimination, with an AUC of 0.83 (Fig. 2). Table 5 shows the sensitivity and specificity at different cutoff scores. The best cutoff score to detect insomnia was 4.5, with a sensitivity of 0.81 and specificity of 0.70. As the global score of the PSQI must be an integer, a score of 5 was chosen as the best cutoff.

**Table 5:** Various cutoff scores for the Chinese version of the PSQI

Cutoff score	Sensitivity	Specificity
0.5	1.00	0.03
1.5	1.00	0.11
2.5	1.00	0.28
3.5	0.92	0.49
4.5	0.81	0.70
5.5	0.66	0.82
6.5	0.53	0.91
7.5	0.32	0.94
8.5	0.15	0.97
9.5	0.10	0.98
10.5	0.09	0.99
13.0	0.03	1.00
16.0	0.00	1.00

## Discussion

Although sleep disruption is prevalent in pediatric patients after completion of cancer treatment, no validated instrument is available to assess this symptom. To address this lack, we translated and adapted the PSQI, which is commonly used among adult cancer patients, for Hong Kong Chinese cancer survivors. We also examined the psychometric properties of the Chinese version of the PSQI in this population.

Consistent with previous studies of young people and community-based adolescents [19, 20], the Chinese version of the PSQI demonstrated acceptable internal consistency; corrected item–total correlations ranged from 0.27 to 0.63. This indicated that all items in the translated PSQI measure the same construct: sleep quality. Additionally, the ICC at 2 weeks was 0.90. This is in accordance with previous studies showing that the PSQI had good stability in measuring subjective sleep quality [19, 20].

Concerning the content validity, most items reflected the underlying construct of sleep quality. Nevertheless, we changed the wording of item 8 with reference to its low CVI. In the original version, item 8 asks an individual to report how often he or she had trouble staying awake under different scenarios, including driving. However, this scenario was not relevant to our target population because they were not able to drive (the legal driving age in Hong Kong is 18 years). Schools play an important role in child and youth development [46]. Therefore, our expert panel changed “driving” to “doing homework” to make item 8 more appropriate for our population.

We examined the construct validity of the Chinese version of the PSQI using the known-group technique. In line with some previous studies [6, 47], our results indicated that survivors of childhood cancer had a higher mean PSQI score than their healthy counterparts, but a lower mean score than those undergoing cancer treatment. This confirmed that the translated PSQI was able to differentiate the sleep quality of different groups of children.

It is well-documented that sleep is one of the most effective ways for the body to restore energy [48]. Inadequate sleep therefore leads to fatigue and affects daily activities [10, 11], resulting in depression and lower quality of life [12, 13]. Consistent with existing literature, we found a negative correlation between PSQI and PedsQL scores. Positive correlations were observed between PSQI and CES-DC scores, and between PSQI and FS-C/FS-A scores, indicating that the Chinese version of the PSQI showed convergent validity.

There is no consensus on the dimensionality of the PSQI [49]. Buysse et al. [15] suggested that the seven components of the PSQI should be combined into a single factor. Cole et al. [26] identified a three-factor model comprising sleep efficiency, sleep quality, and daily dysfunction. Magee et al. [27] proposed a two-factor model because they found an extraordinarily high correlation between the factors of sleep quality and daily disturbance, suggesting that these two factors overlap. The present findings are consistent with those of Cole et al. [26] in that the three-factor model achieved a better fit than the single- and two-factor models. We also observed a reasonable correlation between the factors of sleep quality and daily disturbance, thus confirming that they are two different constructs. All these findings provide empirical evidence that our translated PSQI can assess the sleep quality of survivors of childhood cancer in terms of three separate domains. Merz and Tomfohr-Madsen [6] considered sleep problems in childhood cancer survivors as multidimensional, and conceptualized them as a range of sleeping difficulties related to biological and psychosocial aspects. Our translated PSQI could be used to detect sleep problems located on only one of the three factors. Appropriate interventions could therefore be chosen according to the type and nature of the sleep problem.

The confirmatory factor analysis showed that most components achieved high factor loadings on the three-factor model, except component 6 (the use of sleep medication obtained a factor loading of 0.48). This result is similar to findings from other PSQI validation studies in the Chinese population [50]. A possible explanation is that Chinese people do not commonly use sleep medication because they often believe that it has many side effects [51]. Instead, they tend to use complementary and alternative approaches (e.g., acupuncture and aromatherapy) to relieve sleep problems [52, 53]. This explanation is supported by our findings: 97% of subjects answered “not during the past month” when responding to item 7 on the PSQI: “how often have you taken medicine (prescribed or over-the-counter) to help you sleep?” In response to this issue, we reran the confirmatory factor analysis by removing this component score from our models. However, this did not greatly improve the fit indices. Future studies should consider examining the role of this component in assessing sleep quality in other pediatric patients.

The confirmatory factor analysis also showed that the use of sleep medication was accounted for by the latent variable of sleep efficiency. This is different from the three-factor model proposed by Cole et al. [26], in which the use of sleep medication was accounted for by the latent variable of sleep quality. Such inconsistency may be because people with sleep problems may take medications for various reasons, such as trouble falling asleep (associated with sleep quality) and short sleep duration (related to sleep efficiency). Hence, the use of medications is a poor indicator of latent variables [54].

The results showed that some factor loadings in the three-factor model were high and equal. This implied a certain amount of overlapping covariance contributing to the factors. As such, we have tried to rerun the model by dropping some variables. However, the factor loadings were still similar. Since previous literature suggests that all these variables are important to assess subjective sleep quality [26], we therefore decided to retain all the variables in the confirmatory factor analysis model notwithstanding the overlapping covariance.

Confirmatory factor analysis has also been separately performed on children (aged 6 to 12) and adolescents (aged 13 to 18) as subgroups. The same factor structure with similar factor loadings was shown, except a lower factor loading in the item relating to the number of hours of sleep in adolescents than in children. A possible explanation is that adolescents require shorter duration of sleep than children biologically [55]. Hence the number of hours of sleep in adolescents is less associated with sleep quality when compared with children, resulting in a lower factor loading.

The ROC analysis results revealed that a global score of ≥ 5 was the best cutoff score for distinguishing survivors of childhood cancer with and without insomnia under the diagnostic criteria of DSM-5. The AUC was 0.83, which indicated that the Chinese version of the PSQI has acceptable discrimination. Although our identified cutoff score was slightly lower than that in university students [56], different PSQI cutoff scores have been reported in different studies, according to sample characteristics [15, 57–60].

One of the main strengths is the originality of the research question. Our study bridged an existing research gap by translating the PSQI from English into Chinese and examining the psychometrics of the translated scale among childhood cancer survivors. Another strength is that our study was conducted in Queen Mary Hospital, the main hospital in Hong Kong that provided medical consultation for pediatric oncology patients. Hence, the sample was representative.

### Limitations

A major limitation is that the limited budget precluded use of objective sleep measures, such as actigraphy, to validate PSQI results. Another limitation is that although polysomnography (PSG) is regarded as the gold standard to diagnose insomnia, we only used DSM-5 as the diagnostic tool for screening participants with insomnia since the use of PSG is not a routine clinical procedure and is highly complicated with laboratory analysis. The third limitation is that we did not perform a formal assessment to measure the residual function of our participants. However, since they were required to complete the questionnaires on their own, it is expected that their residual function of cognitive domains should be comparable to that of healthy children. Hence, our results might not be generalizable to childhood cancer survivors with low residual function. The fourth limitation is that we did not compare the data collected from the telephone version of the translated PSQI with that from face-to-face. Hence, we cannot conclude the validity of the telephone version.

## Conclusion

There is increasing evidence of the serious effects of sleep disruption on survivors of childhood cancer [9–12]. However, the problem is not routinely assessed, which indicates room for improvement in survivorship care [61]. The validated PSQI could be used in clinical settings to monitor the subjective sleep quality of survivors of childhood cancer. This would help to provide early assessments and interventions to alleviate sleep disruption and minimize its associated long-term healthcare cost. Previous systematic reviews have identified different effective interventions to minimize sleep disruption among adult cancer patients [62, 63]. However, owing to a lack of validated instruments to assess sleep quality among childhood cancer survivors, these interventions have never been tested in this populations. It thus remains unclear whether they are useful for survivors of childhood cancer. The validated PSQI could be used to evaluate the effectiveness of these interventions to reduce sleep disruption among childhood cancer survivors.

This study examined the psychometric properties of the Chinese version of the PSQI. The translated PSQI was demonstrated to be a reliable and valid instrument to assess subjective sleep quality. The three-factor structure of the PSQI proposed by previous studies was confirmed.

## Data Availability

The data will be available upon reasonable request.

## Abbreviations

PSQI: Pittsburgh Sleep Quality Index
CES-DC: Center for Epidemiologic Studies Depression Scale for Children
FS-C: Fatigue Scale-Child
FS-A: Fatigue Scale-Adolescent
PedsQL 4.0: Pediatric Quality of Life Inventory 4.0 Generic Core Scale
CVI: Content validity index
I-CVI: Item content validity index
S-CVI: Scale content validity index
*df*: Degrees of freedom
RMSEA: Root mean square error of approximation
CFI: Comparative fix index
ROC: Receiver operating characteristic
DSM-5: Diagnostic and Statistic Manual of Mental Disorders Fifth Edition
AUC: Area under the curve
PSG: Polysomnography
